# Evaluation of Microbiological Performance and the Potential Clinical Impact of the ePlex^®^ Blood Culture Identification Panels for the Rapid Diagnosis of Bacteremia and Fungemia

**DOI:** 10.3389/fcimb.2020.594951

**Published:** 2020-11-26

**Authors:** Sabrina Bryant, Iyad Almahmoud, Isabelle Pierre, Julie Bardet, Saber Touati, Daniele Maubon, Muriel Cornet, Claire Richarme, Max Maurin, Patricia Pavese, Yvan Caspar

**Affiliations:** ^1^Laboratoire de bactériologie-hygiène hospitalière, Centre Hospitalier Universitaire Grenoble Alpes, Grenoble, France; ^2^Univ. Grenoble Alpes, CNRS, CHU Grenoble Alpes, Grenoble INP, TIMC-IMAG, Grenoble, France; ^3^Service des maladies infectieuses et tropicales, Centre Hospitalier Universitaire Grenoble Alpes, Grenoble, France

**Keywords:** sepsis, bacteremia, fungemia, early diagnosis, multiplex polymerase chain reaction

## Abstract

Molecular rapid diagnostic assays associated with antimicrobial stewardship have proven effective for the early adaptation of empiric therapy in bloodstream infections. The ePlex^®^ BCID (GenMark Diagnostics) Panels allow identification of 56 bacteria and fungi and 10 resistance genes in 90 min directly from positive blood cultures. We prospectively evaluated 187 sepsis episodes at Grenoble University Hospital and retrospectively analyzed the cases to measure the potential clinical impact of the ePlex BCID results. Identification of all pathogens was obtained for 164/187 (88%) bloodstream infections with 100% detection of antimicrobial resistance genes (17 *bla_CTX-M_*, 1 *vanA*, and 17 *mecA* genes). Only 15/209 (7%) strains were not covered by the panels. Sensitivity for detection of micro-organisms targeted by the RUO BCID-GP, BCID-GN, and BCID-FP Panels was respectively 84/84 (100%), 103/107 (96%), and 14/14 (100%). Interestingly, accurate identification of all pathogens was achieved in 15/17 (88%) polymicrobial samples. Retrospective analysis of medical records showed that a modification of antimicrobial treatment would have been done in 45% of the patients. Treatment modifications would have been an optimization of empiric therapy, a de-escalation or an escalation in respectively 16, 17, and 11% of the patients. Moreover, 11% of the samples were classified as contaminants or not clinically relevant and would have led to early de-escalation or withdrawal of any antibiotic. Detection of resistance genes in addition to identification alone increased escalation rate from 4 to 11% of the patients. Absence of the ePlex result was considered a lost opportunity for therapy modiﬁcation in 28% of patients.

## Introduction

Over 1,700,000 episodes of bloodstream infections (BSI) are diagnosed each year in Europe and North America. They account for more than 230,000 deaths per year with mortality rates between 10 to 40%, largely impacted by the increased burden of bacterial resistance (Goto and Al-Hasan, 2013; Huang et al., 2013; Patel et al., 2017; Martin et al., 2018; Robineau et al., 2018). Early administration of an effective antimicrobial therapy is one of the main factors for survival in cases of sepsis and septic shock (Kumar et al., 2006; Seymour et al., 2017). However, empiric antibiotic therapy remains inappropriate in 20–40% of the patients mainly because there is wide diversity of pathogens and increased prevalence of resistance mechanisms while additionally only one quarter of patients with invasive fungal infections are receiving an adequate and early treatment (Eggimann et al., 2015; Robineau et al., 2018; Ioannidis et al., 2020; Seok et al., 2020).

Once a blood culture has been collected, time to result relies on time to positivity of the blood culture and on time to obtain identification and antimicrobial susceptibility testing (AST) data from the positive bottles. Time to positivity is influenced by sampling volumes, growth rate of the bacteria or fungi, transportation time and delay of bottle loading in the incubators (Banerjee et al., 2016; Lamy, 2019). Time to obtain identification and AST results depends on the microbiological diagnostic tools used by each laboratory. Several innovative methods have been developed to shorten this time to result for microorganism identification in positive blood culture bottles. MALDI-TOF mass spectrometry (MALDI-TOF-MS) provides rapid identification at a low cost. However, this technology requires skilled lab staff, includes several manual steps that may prohibit the ability to process the positive blood cultures on a 24/7 basis and poorly identifies pathogens in polymicrobial samples (Fiori et al., 2016; Dubourg et al., 2018; Lamy et al., 2020). On the other hand, molecular methods are costlier and limited to a reduced number of pathogens, but they also detect antimicrobial resistance genes with high clinical impact to better guide fast adaptation of appropriate antimicrobial therapy. In a large meta-analysis, molecular rapid diagnostic assays have shown decreased mortality and length of stay in hospitals when associated with antimicrobial stewardship (Timbrook et al., 2017). Quick antibiotic susceptibility results may be obtained several minutes or hours later by other methods (i.e. Rapid AST, MS-based assays, colorimetric rapid tests, etc.) requiring additional manual steps and costs while delaying the result (Dubourg et al., 2018; Vatanshenassan et al., 2018; Lamy et al., 2020). This rapid reporting for the identification of bacteria or fungi along with antimicrobial resistance gene data by the laboratory in combination with antimicrobial stewardship allows accelerated replacement of ineffective or non-optimal therapies with active antibiotics or antifungals with an optimized efficacy against the microorganism(s) identified. Moreover, rapid detection of the absence of some important resistance mechanism(s) may allow de-escalation of treatment and thus reduce the use of broad spectrum (i.e. carbapenems) or toxic (vancomycin) antibiotics (Huang et al., 2013; Reed et al., 2014; Banerjee et al., 2015; MacVane and Nolte, 2016; Patel et al., 2017; Timbrook et al., 2017).

The ePlex system (GenMark Diagnostics, Carlsbad, CA) is a random access multiplex PCR platform developed for syndromic diagnosis. Three Blood Culture Identification (BCID) Panels have been designed to detect 56 pathogens responsible for BSI and 10 antimicrobial resistance genes in ~90 min. The BCID-GN Panel detects 21 Gram-negative (GN) bacterial genera or species and 6 extended-spectrum beta-lactamase (ESBL) or carbapenemase resistance genes (*bla_CTX-M_, bla_OXA_, bla_VIM_, bla_NDM_, bla_KPC_, bla_IMP_*) while the BCID-GP Panel identifies 20 Gram-positive (GP) bacterial genera or species and 4 resistance genes (*mecA, mecC, vanA, vanB*) and the BCID-FP (Fungal Pathogen) Panel detects 15 fungal genera or species (**Table S1**).

At the time of the study, only the Research Use Only (RUO) cartridges were available, but all panels are now FDA-Cleared and CE-IVD marked. The aim of this study was to prospectively evaluate the microbiological performance of the ePlex BCID Panels and retrospectively analyze their potential clinical impact in our university hospital.

## Material and Methods

### Prospective Study of Microbiological Performance

The study was performed at Grenoble University Hospital, a major teaching hospital (2,100 beds) serving a regional population of 670,000 inhabitants and covering the following specialties: intensive care, general medicine, surgery, geriatric and pediatric medicine, transplants, and oncology. Between November 2016 and November 2017, we prospectively analyzed 187 BSI episodes. Depending on the availability of the RUO cartridges, all first positive blood cultures (BD Bactec Plus Aerobic/F, Lytic/10 Anaerobic/F or Peds Plus/F, Becton Dickinson, Pont de Claix, France) were tested on a given day to avoid selection biases. We included only the first positive blood culture for each BSI episode after microscopic confirmation to exclude false positive bottles and to select the appropriate ePlex Panel(s) to run (one BCID-GP, GN, FP or several panels if Gram stain revealed a polymicrobial sample). We did not exclude suspicion of contaminants. The study design was non-interventional and the results were not reported to the clinicians or used for the management of the patients.

### Standard Microbiological Procedure for BSI Diagnosis

**Figure 1:**
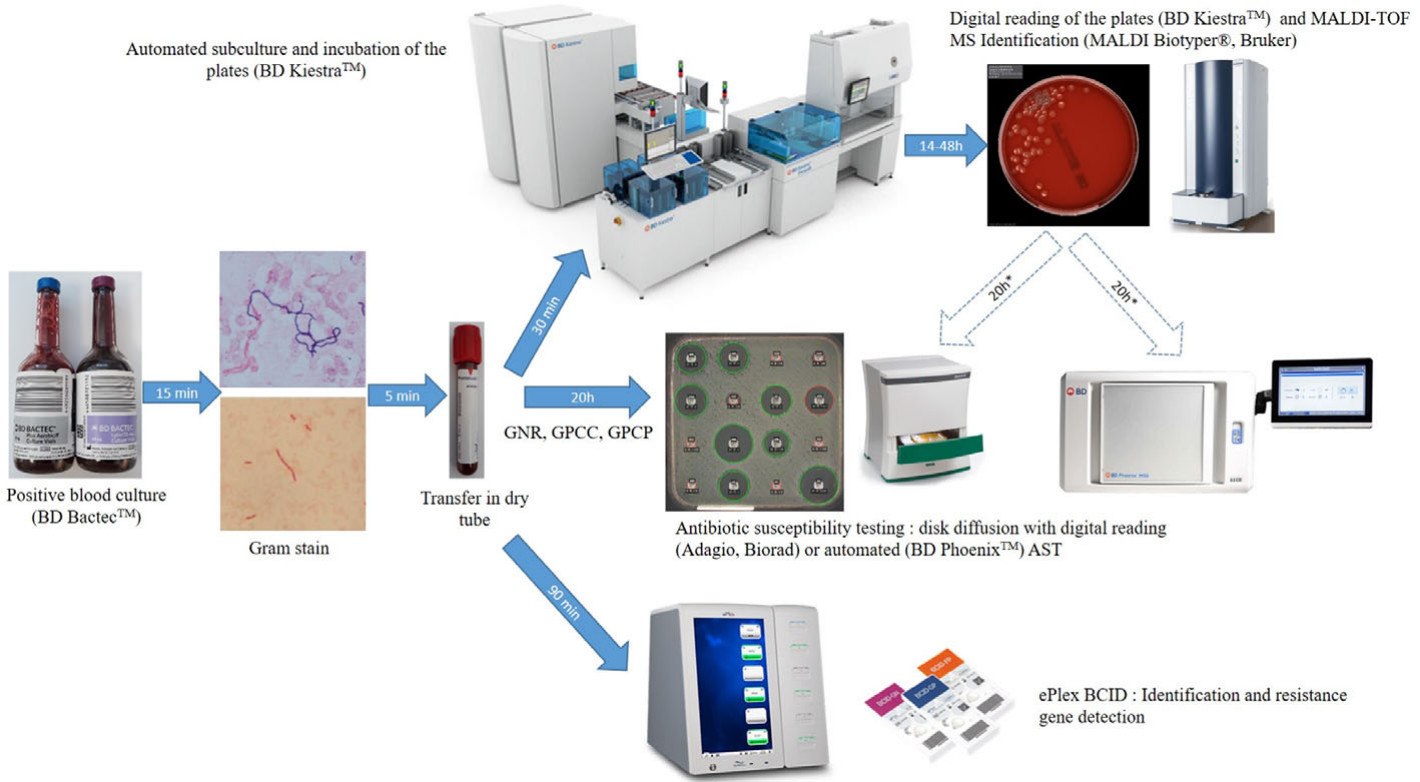
Workflow of ePlex BCID Panels and standard of care testing of positive blood cultures. GNR, Gram-negative rods; GPCC, Gram-positive cocci in cluster; GPCP, Gram-positive cocci in pairs or chains.

Standard-of-care (SOC) testing of positive blood cultures was conducted as follows. Positive blood cultures detected by the BD Bactec™ from 8:00 to 23:00 were confirmed by microscopic examination after Gram staining (**Figure 1**). An aliquot of positive blood culture was transferred into a dry tube (BD Vacutainer) and subcultured automatically by streaking non-selective (based on the Gram stain result: one 5% sheep blood Columbia or Polyvitex agar for aerobic incubation and one for anaerobic incubation; BioMérieux, Marcy l’Etoile, France) and selective media (if required based on the Gram stain: Drigalski medium, 5% sheep blood Columbia CNA agar or CAN2 medium for fungus) using the automated BD Kiestra™ Work Cell Automation (WCA) or manual streaking. Drigalski and CAN2 media were automatically incubated under ambient atmosphere whereas Columbia blood agar and Polyvitex agar were automatically incubated under a 5% CO2 enriched atmosphere in the connected incubators of the WCA. The anaerobic media were incubated in anaerobic jars. The remaining dry tube filled with positive blood culture was then used for ePlex testing and subsequently frozen at -80°C for future resolution of discrepancies. Automated digital reading of the agar plates was performed using BD Kiestra™ WCA at 14, 24, and 48 h of incubation. Colonies were identified after 14 to 48 h of growth by MALDI-TOF mass spectrometry on the Microflex LT instrument (Bruker Daltonics, Bremen, Germany) using IVD-MBT software (version 6, 6763 MSP in the database) after addition of 1 µl formic acid if required, as recommended by the manufacturer.

AST using disk diffusion method was performed directly from bacterial positive blood cultures using CASFM-EUCAST guidelines: Mueller Hinton agar incubated under ambient atmosphere for Gram-positive cocci in cluster (GPCC) and Gram-negative bacilli or on 5% horse blood Mueller Hinton-F agar with 20 mg/L β-NAD (MHE and MH-F medium respectively, BioMérieux) incubated under a 5% CO2 enriched atmosphere for Gram-positive cocci in pairs or chains (GPCP). If a GPCP was suspected by microscopic examination, an additional 5% sheep Columbia blood agar was streaked before adding an optochin disk (Biomérieux) and the agar plate was incubated under a 5% CO2 enriched atmosphere. If other bacteria were suspected, disk diffusion was performed only after subculture of the positive blood culture, on isolated colonies. If the bacterial layer of AST media was not confluent, or if the panel tested was incorrect or extensive testing was required, an additional antibiogram could be performed the next day on isolated colonies by the automated BD Phoenix system (Becton Dickinson, Pont de Claix, France) using PMIC-75 card for *Staphylococcus* and *Enterococcus* strains and NMIC-93 for GN bacilli or by disk diffusion methodology for other bacterial genera. Confirmation of ESBL production or of hyperexpression of the *ampC* gene was done phenotypically using synergy testing and restoration of third generation cephalosporin activity in the presence of cloxacillin following CASFM-EUCAST guidelines. Presence of a *vanA* or *vanB* gene was performed using the Xpert *vanA*/*vanB* assay on the GeneXpert instrument (Cepheid, Sunnyvale, CA, USA) in cases under suspicion of a vancomycin-resistant enterococci. Resistance to methicillin in Staphylococcal species was inferred from resistance to a cefoxitin disk. Antifungal susceptibility testing was performed using E-test MIC strips (Biomérieux).

### Testing of Positive Blood Cultures With the ePlex BCID Panels

Positive blood culture samples were tested from 9:00 to 18:00 as soon as possible and always within 12 h of positivity as recommended by the manufacturer. Briefly, a 50 µl aliquot of the blood sample from a positive blood culture in the dry tube was dispensed using a micropipette into the appropriate cartridge (BCID-GN, GP, and/or FP) according to Gram stain results. The cartridge was scanned at the barcode reader, inserted into the ePlex instrument and results were available in ~90 min. The BCID-GP and BCID-GN Panels also contain 2 Pan targets each (BCID-GP: Pan Gram-Negative, Pan *Candida*; BCID-GN: Pan Gram-Positive, Pan *Candida*) to aid in the detection of polymicrobial organisms and organisms that may produce inaccurate or misleading Gram stain results. If an unsuspected Pan target was detected, we analyzed the sample with the corresponding ePlex BCID Panel.

### Retrospective Analysis of Potential Clinical Impact

**Figure 2:**
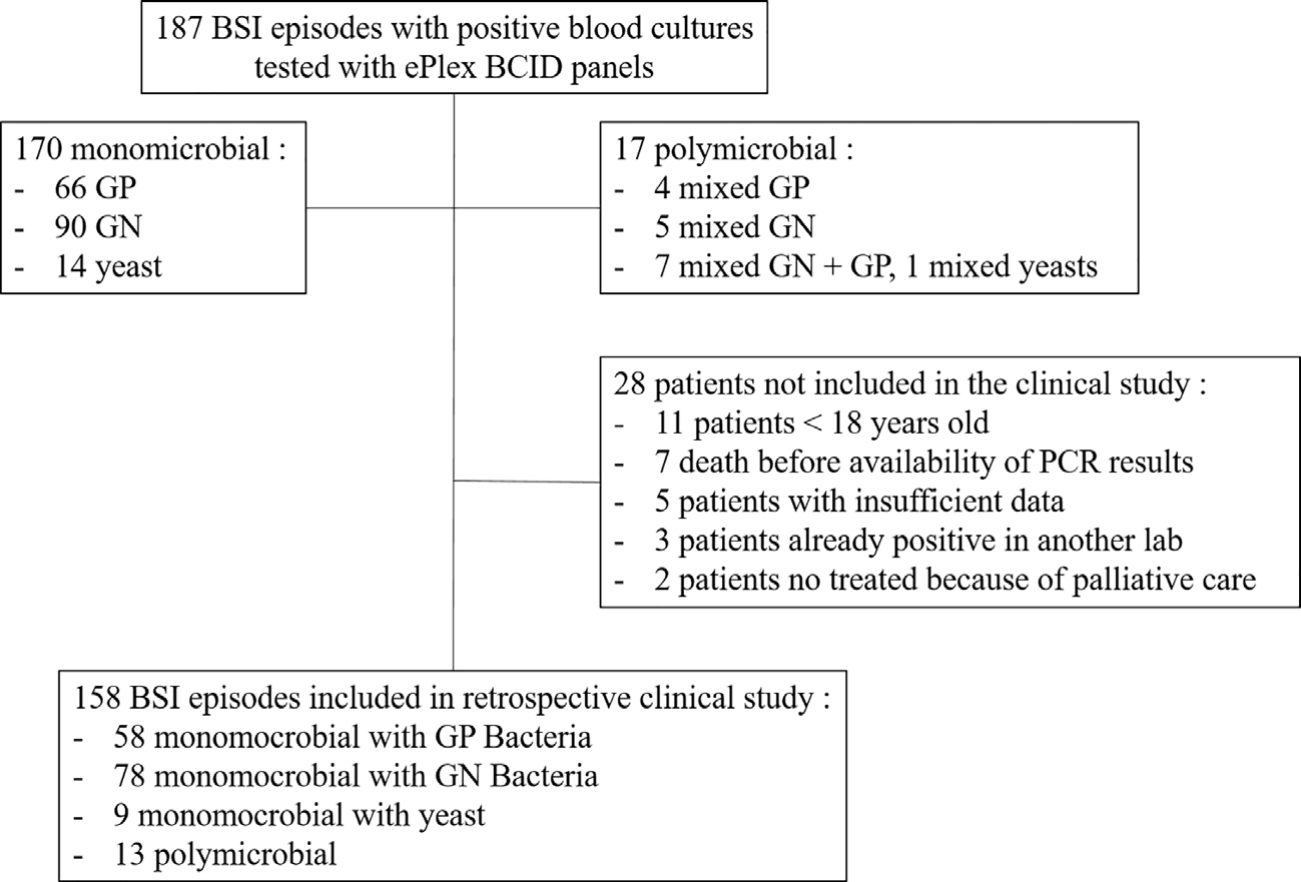
Flow chart of the prospective performance study and of the retrospective clinical study.

Clinical data of BSI episodes from patients ≥18 years old for whom a positive blood culture had been evaluated by the ePlex BCID Panels was collected retrospectively using an electronic case report form (eCRF). Patients were not included in the analysis if they had died before the PCR result would have been available, if insufficient data were available, if results of a prior positive blood culture from another laboratory was available or for patients in which no antibiotic therapy was started 24 h after blood culture positivity because of palliative care (**Figure 2**). Complete medical history of the BSI episode was then reviewed during a multidisciplinary meeting with at least two infectious disease physicians and one microbiologist member of the antibiotic stewardship team for the management of BSI in our hospital. For each episode of BSI, they had to determine if the early results of the ePlex BCID Panel(s) would have led to modifications in the management of the patients: 1) would the result of identification of the bacteria or fungus have led to a modification of antibiotic or antifungal treatment; 2) would the result of antimicrobial resistance genes have led to a modification of antibiotic or antifungal treatment. For items 1 and 2, participants were asked to stratify their answers as optimization/escalation/de-escalation/stop of the antimicrobial treatment; 3) would the results of the ePlex BCID Panel(s) alone have led to an erroneous decision; 4) would the results of the ePlex BCID Panel(s) combined to the previous result of the Gram stain have led to an erroneous decision; 5) was the absence of ePlex BCID Panel(s) a loss of opportunity for patient management; 6) would the result of the ePlex BCID Panel(s) have led to other modifications in the management of the BSI episode (such as infection control measures, catheter removal, etc.). For items 3 to 6, participants were asked to stratify their answers as yes/probably/probably not/no. Escalation corresponded to the start of an effective therapy if none had been started or to broadening the bacterial spectrum of the antibiotic therapy. De-escalation was defined as narrowing of the antibacterial spectrum of the antibiotic therapy or the stop of at least one antibiotic. Optimization was considered when another antibiotic class or subclass would have been used for better efficacy. No nominative or sensitive personal data have been collected and the study only involved the reuse of already available data. Ethical approval has been obtained based on French legislation. This study falls within the scope of the French Reference Methodology MR-004 for studies involving human samples and medical data obtained during routine diagnostic procedures. An informative letter has been sent to each participant to check that they do not oppose participation in the study.

### Statistical Analyses

Results obtained with the ePlex BCID Panels were compared to results obtained with SOC methods. We calculated sensitivity and specificity for each target using the numbers of True positive, False positive, True negative, and False negative results (TP, FP, TN and FN) as follows: Se (%) = 100 x TP/(TP+FN) and Sp = 100 x TN/(TN+FP). A TP result for a given target was obtained when SOC confirmed identification or resistance mechanism provided by an ePlex BCID Panel. Due to the fact that the study evaluated positive blood cultures only, a TN was considered a result from the ePlex with no bacterial, fungal or antimicrobial resistance gene targets detected correlating with a SOC result that showed absence of growth for any pathogen with no potential corresponding resistance mechanism that would be present on an ePlex BCID Panel. A FP result corresponded to the identification of a particular target by an ePlex BCID Panel not confirmed by SOC testing. A FN corresponded to the absence of detection of a target present on the ePlex BCID Panels when the microorganism grew or the resistance mechanism was detected by SOC testing.

### Analysis of Discrepancies

Discrepancies were analyzed in light of all positive blood cultures for a given BSI episode. FN results were tested again by culture using frozen aliquots and using the ePlex. A FP sample could eventually be switched to a TP if the bacteria or fungi detected by an ePlex BCID Panel grew in another blood culture bottle from the same BSI episode. Overall agreement for each sample (agreement of all identification and antimicrobial resistance gene targets present in a given sample) was determined after resolution of the potential discrepancies. As RUO cartridges were tested initially, retesting of discrepant samples after modifications of thresholds and parameters of the test by the manufacturer was performed on FDA approved/CE-IVD marked cartridges from frozen samples.

## Results

### Performance of the ePlex BCID Panels for Monomicrobial BSI

Overall, 187 BSI episodes were analyzed by the ePlex BCID Panels (**Figure 2**). Comparison of the results to SOC testing for the ePlex BCID-GP, BCID-GN, and BCID-FP Panels are gathered in **Tables 1**–**3** respectively. With regard to monomicrobial BSI, no false-negative results were obtained for the 62 GP bacterial strains and for the 12 *Candida* spp. strains for which a target was present on the BCID-GP Panel or the BCID-FP Panel, respectively. Only 4/66 (6%) GP bacteria in monomicrobial samples and 2/14 (14%) yeasts were not targeted by the corresponding panel. On the other hand, the BCID-GN Panel detected 89/90 (99%) GN isolates for which a target was defined. One strain of *Pseudomonas aeruginosa* was not identified and only 5/90 (6%) GN bacteria in monomicrobial samples were not targeted by this panel. Four false-positive results were obtained with both the BCID-GP and BCID-GN Panel. Analysis of discrepancies are presented in **Table 4**. All these false positive results were never confirmed by standard culture even after repeating the culture from frozen samples or by running the panel corresponding to the false positive Pan target identified.

**Table 1:** Results and performance of bacterial identification and antimicrobial resistance gene detection using ePlex BCID-GP panel compared to standard of care results.

BCID-GP Panel Targets (n = 77)	Identification and resistance results by SOC testing	Se (%)	Sp (%)
*Staphylococcus aureus*	*S. aureus*	18/18 (100)	59/59 (100)
*Staphylococcus epidermidis*	*S. epidermidis*	12/12 (100)	65/65 (100)
*Staphylococcus lugdunensis*	*S. lugdunensis*	1/1 (100)	76/76 (100)
*Staphylococcus*	*S. hominis* (8); *S. capitis* (2); *S. haemolyticus* (1)	42/42 (100)	35/35 (100)
*Enterococcus faecalis*	*E. faecalis*	7/7 (100)	70/70 (100)
*Enterococcus faecium*	*E. faecium*	7/7 (100)	70/70 (100)
*Enterococcus*	None other species	14/14 (100)	63/63 (100)
*Streptococcus pneumoniae*	*S. pneumoniae*	4/4 (100)	73/73 (100)
*Streptococcus pyogenes*	*S. pyogenes*	1/1 (100)	76/76 (100)
*Streptococcus agalactiae*	None		77/77 (100)
*Streptococcus anginosus group*	*S. milleri* group	4/4 (100)	73 (100)
*Streptococcus*	*S. mitis/oralis* group (4); *S. gallolyticus* (2); *S. sanguinis* (1); *S. parasanguinis* (2)	18/18 (100)	59/59 (100)
*Corynebacterium*	*C. striatum*	1/1 (100)	76/76(100)
*Micrococcus*	None		77/77 (100)
*Lactobacillus*	None		77/77 (100)
*Bacillus cereus* group	None		77/77 (100)
*Bacillus subtilis* group	*B. subtilis* group	1/1 (100)	76/76(100)
*Cutibacterium acnes*	*C. acnes*	1/1 (100)	76/76(100)
Pan Gram-Negative	*P. aeruginosa* (2); *B. fragilis* (2); *E. coli* (1); *P. mirabilis* (2)	7/7 (100)	68/70 (97)
Pan *Candida*	None		75/77 (97)
*mecA* gene	*S. aureus* (2); *S. epidermidis* (9); *S. hominis* (5); *S. haemolyticus* (1)	17/17 (100)	60/60 (100)
*vanA* gene	*E. faecium* (1)	1/1 (100)	76/76 (100)
*vanB* gene			77/77 (100)
No Targets Detected	*Gordonia sp* (1); *Aerococcus viridans* (1); *Finegoldia magna* (1); *Bacillus sp* (1); *Collinsella aerofaciens* and *Dialister pneumosintes* (1); *Actinomyces neuii* and *Veillonella parvula* (1)		

**Table 2:** Results and performance of bacterial identification and antimicrobial resistance gene detection using ePlex BCID-GN panel compared to standard of care results.

BCID-GN Panel Targets (n = 102)	Identification and resistance results by SOC testing	Se (%)	Sp (%)
*Escherichia coli*	*E. coli*	57/57 (100)	45/45 (100)
*Klebsiella pneumoniae*	*K. pneumoniae*	12/12 (100)	90/90 (100)
*Klebsiella oxytoca*	*K. oxytoca*	3/3 (100)	99/99 (100)
*Proteus mirabilis*	*P. mirabilis*	5/5 (100)	97/97 (100)
*Proteus*	No other species	5/5 (100)	97/97 (100)
*Enterobacter cloacae* complex	*E. cloacae* complex	1/1 (100)	100/101 (99)
*Enterobacter* (non-*cloacae* complex)	None		102/102 (100)
*Citrobacter sp*	*C. koseri*	3/3 (100)	99/99 (100)
*Serratia marcescens*	*S. marcescens*	3/3 (100)	99/99 (100)
*Serratia*	No other species	3/3 (100)	99/99 (100)
*Morganella morganii*	*M. morganii*	1/1 (100)	101/101 (100)
*Cronobacter sakazakii*	None		102/102 (100)
*Salmonella sp*	*S. enterica* serotype Dublin	1/1 (100)	101/101 (100)
*Pseudomonas aeruginosa*	*P. aeruginosa*	6/8 (75)	94/94 (100)
*Acinetobacter baumanii*	None		102/102 (100)
*Stenotrophomonas maltophilia*	None		99/102 (97)
*Haemophilus influenzae*	*H. influenzae*	3/3 (100)	99/99 (100)
*Neisseria meningitidis*	None		101/102 (99)
*Bacteroides fragilis*	*B. fragilis*	5/5 (100)	97/97 (100)
*Fusobacterium nucleatum*	None		102/102 (100)
*Fusobacterium necrophorum*	None		102/102 (100)
Pan *Candida*	None		102/102 (100)
Pan Gram-Positive	*Streptococcus anginosus* group (2); *E. faecium* (2); *E. faecalis* (1)	3/5 (60)	96/97 (99)
*bla_CTX-M_* gene	*E. coli* (11); *K. pneumoniae* (5); *S. marcescens* (1)	17/17 (100)	85/85 (100)
*bla_OXA_, bla_VIM_, bla_NDM_, bla_KPC_, bla_IMP_*	None		102/102 (100)
No Targets Detected	*Acinetobacter johnsonii* (1); *Acinetobacter pitii* (1); *Paracoccus sanguinis* (1); *Pantoea agglomerans* (1); *Aeromonas hydophila* (1), *Collinsela aerofaciens* + *Dialister pneumosintes* (1), *C. acnes* (1)		

**Table 3:** Results and performance of fungal identification using ePlex BCID-FP panel compared to standard of care results.

BCID-FP Panel Targets (n = 15)	Identification and resistance results by SOC testing	Se (%)	Sp (%)
*Candida albicans*	*C. albicans*	5/5 (100)	10/10 (100)
*Candida glabrata*	*C. glabrata*	3/3 (100)	12/12 (100)
*Candida parapsilosis*	*C. parapsilosis*	2/2 (100)	13/13 (100)
*Candida guilliermondii*	*C. guilliermondii*	2/2 (100)	13/13 (100)
*Candida kefy*	*C. kefyr*	1/1 (100)	14/14 (100)
*Candida krusei*	*C. krusei*	1/1 (100)	14/14 (100)
Other targets (*Candida auris, Candida dubliniensis, Candida famata, Candida lusitaniae, Candida tropicalis, Cryptococcus gattii, Cryptococcus neoformans, Fusarium, Rhodotorula*)	None		15/15 (100)
No Target Detected	*C. orthopsilosis* (1); *C. inconspicua* (1)		

**Table 4:** Analysis and resolution of discrepancies between ePlex BCID Panel results and standard culture.

Gram stain result	ePlex RUO BCID Panel and results	Primary culture results	Secondary culture results if different	Results of ePlex Panel (positive Pan target)	ePlex CE-IVD BCID Panel re-testing	Conclusion
GPCC	BCID-GP: *S. aureus*, *mecA*, **Pan *Candida***	MR *S. aureus*		BCID-FP: no target detected	*S. aureus, mecA*	False positive
GPCC	BCID-GP: *S. epidermidis*, **Pan *Candida***	*S. epidermidis*		BCID-FP: no target detected	*S. epidermidis*	False positive
GPCC	BCID-GP: *S. epidermidis, mecA*, **Pan Gram-Negative**	MR *S. epidermidis*		BCID-GN: no target detected*	*S. epidermidis, mecA*	False positive
GPCP	BCID-GP: *S. pneumoniae*, **Pan Gram-Negative**	*S. pneumoniae*		BCID-GN: no target detected*	*S. pneumoniae*	False positive
EB-GNR	BCID-GN: *E. coli*, ***S. maltophilia, N. meningitidis***	*E. coli*		None performed	*E. coli*	False positive
EB-GNR	BCID-GN: *E. coli*, ***E. cloacae complex***	*E. coli*		None performed	*E. coli*	False positive
EB-GNR	BCID-GN: *K. oxytoca*, **Pan Gram-Positive**	*K. oxytoca*		BCID-GP: no target detected	*K. oxytoca*	False positive
GNR	BCID-GN: *C. koseri*, ***S. maltophilia***	*C. koseri*		None performed	*C. koseri*	False positive
GNR	BCID-GN: No target detected	*P. aeruginosa*		None performed	*P. aeruginosa*	False negative
GNR	BCID-GN: *P. aeruginosa*, ***S. maltophilia***	*P. aeruginosa*	*+ S. maltophilia*	None performed	*P. aeruginosa, S. maltophilia*	True positive
EB-GNR and GPCP	BCID-GP: *S. anginosus* group,Pan Gram-Negative BCID-GN: *P. mirabilis* **(Pan Gram-Positive not detected)**	*S. anginosus* group, *P. mirabilis*		None performed	BCID-GN: *Proteus mirabilis*, Pan Gram-Positive	False negative
Aerobic BC: GNR Anaerobic BC: short GNR and GPCP	Aerobic BC: BCID-GN: *B. fragilis*,**(*P. aeruginosa* not detected)** Anaerobic BC: BCID-GP: Pan Gram-Negative BCID-GN: *B. fragilis, P. aeruginosa*	Aerobic BC: *P. aeruginosa* Anaerobic BC: *B. fragilis,C. aerofaciens, D. pneumosintes*		None performed	Aerobic BC: BCID-GN: *B. fragilis, P. aeruginosa*	False negative of*P. aeruginosa* target in aerobic BC
EB-GNR and GPCP	BCID-GP: *Enterococcus, E. faecium*, Pan Gram-Negative BCID-GN: *E. coli, bla_CTX-M_* **(Pan Gram-Positive not detected)**	*E. faecium*, ESBL-producing *E. coli*		None performed	BCID-GN: *E. coli, bla_CTX-M_*, Pan Gram-Positive	False negative
GPCP	BCID-GP: *Streptococcus, S. anginosus* group, Pan Gram-Negative BCID-GN: ***B. fragilis***, Pan Gram-Positive	*S. anginosus* group	*+ B. fragilis*	None performed	Not performed	True positive
Yeast	Aerobic BC: BCID-FP: *C. krusei, C. glabrata*	Aerobic bottle: *C. krusei* Anaerobic bottle: *C. glabrata*		None performed	Not performed	True positive

*Not all species covered in the Pan Gram-Negative target are covered by the BCID-GN Panel. BC, Blood culture; GNR, Gram-negative rods; EB, Enterobacteriaceae; GPCC, Gram-positive cocci in cluster; GPCP, Gram-positive cocci in pairs or chains.

Discrepancies are indicated in bold.

Underlined values correspond to aerobic and anaerobic positive blood culture bottles from a same pair of blood culture.

### Performance of the ePlex BCID Panels for Polymicrobial BSI

Among the 17 polymicrobial samples, after resolution of discrepancies, detection of all microorganisms was obtained in 15/17 (88%) polymicrobial BSI (**Figure 2** and **Table S2**). One *P. aeruginosa* strain was not detected in the aerobic bottle of a BSI episode in which *Bacteroides fragilis*, *Collinsella aerofaciens* and *Dialister pneumosintes* grew in the anaerobic bottle (**Table 4** and **S2**). In the corresponding anaerobic bottle, the latter two GP species were also not identified by the BCID-GP Panel because they are not targeted by this panel. Similarly, the off-panel organisms *Actinomyces neuii* and *Veillonella parvula* were also not detected in another sample. The 7 mixed GN and GP positive blood cultures were efficiently detected by BCID-GP Panel as the Pan Gram-Negative target was positive for the 7 samples. However only 3/7 Pan Gram-Positive targets were positive on the BCID-GN Panel. Importantly, one *B. fragilis* strain, one *Stenotrophomonas maltophilia* strain and one *Candida glabrata* strain were identified as true-positive in unsuspected polymicrobial samples.

### Performance of the ePlex BCID Panels for the Detection of Antibiotic Resistance Genes

The 17 methicillin-resistant (MR) *Staphylococcus* strains, including 2 MRSA and 15 MR Coagulase-negative *Staphylococcus* strains, and the single vancomycin-resistant *Enterococcus* strain (*vanA*-positive *E. faecium*) isolated during the study, all were all detected by the BCID-GP Panel. The BCID-GN Panel identified a *bla_CTX-M_* gene in 17 strains of *Enterobacterales*. ESBL-production was confirmed in all the strains by synergy assays and no other ESBL-producing strains were identified by SOC testing.

### Concordance With Culture After Resolution of Discrepancies

After resolution of discrepant results, overall concordance (concordance of all targets) of the ePlex BCID-GP, GN, and FP Panels with culture for monomicrobial samples was 62/66 (94%), 85/90 (94%), and 14/14 (100%), respectively and 9/11 (81%), 11/12 (92%), and 1/1 (100%) for polymicrobial samples, respectively. With all samples combined, the sensitivity for detection of bacteria and fungi targeted by the BCID-GP, BCID-GN, and BCID-FP Panels was 84/84 (100%), 103/107 (96%), and 14/14 (100%), respectively. Global identification rate for on-panel organisms detected to the species or genus level was 191/193 (99%). Given that we tested RUO cartridges, slight modifications of thresholds and parameters of the test by the manufacturer were performed before releasing FDA approved/CE-IVD marked cartridges. Retesting of discrepant frozen samples on CE-IVD/FDA approved cartridges eliminated all the false-positive and the false-negative results, increasing overall concordance of all panels to 100%. Including bacteria not targeted by the panels, global identification rate was 164/187 (88%) on the RUO cartridges and increased to 166/187 (89%) after rerun of false-positive and false-negative samples on CE-IVD/FDA approved cartridges.

### High Impact of the Results on Clinical Decisions

Our study retrospectively evaluated potential clinical decisions in 158 adult BSI episodes (**Figure 2** and **Table 5**). Prospective patient inclusion followed by a review of medical records was used to investigate both the diagnostic value and the potential clinical and therapeutic impact of the test. For bacteria, median time to blood culture positivity once loaded into the incubators was 15 h (range: 5–290 h) for the 58 aerobic bottles, 11 h (range: 4–120 h) for the 90 anaerobic bottles and 36 h (range: 25–59 h) for the 10 bottles growing fungus. Potential therapeutic modifications looking based on the ePlex BCID Panel results being available during the BSI episode were discussed through a multidisciplinary meeting of at least 3 senior members of our antibiotic stewardship program. Overall, a modification of antimicrobial treatment would have been performed in 45% of the patients at the time the results of the ePlex BCID Panels would have been available (**Table 4** and **S2**). Treatment modifications would have included an optimization of empiric therapy in 25/158 (16%) patients or a de-escalation in 27/158 (17%). Importantly an escalation would have been possible more rapidly in 17/158 (11%) patients, while another 11% of the samples were classified as contaminants or not clinically relevant, resulting in de-escalation or withdrawal of any antibiotic. We stratified the modification by type of medical unit (**Table 6**) or by Gram stain results (**Table S3**). The data showed that in the ICU and hematology wards the results would have led to an optimization of treatment in 6/23 (26%) and 4/20 (20%) patients respectively or a de-escalation of the therapy in 3/23 (13%) and 4/20 (13%) patients respectively. Escalation of treatment would have occurred for 14/92 (15%) patients in medical wards while optimization would have been possible in 11/92 (12%) patients and de-escalation in 14/92 (15%).

**Table 5:** Characteristics of the patients included in retrospective analysis of potential clinical impact of the ePlex BCID Panel results.

	Bacteremia	Fungemia
BSI (n)	148	10
Sex (M/F)	80/68	6/4
Age (mean +/- SD)	66 +/- 16	61 +/- 18
Medical unit		
ICU	20 (13,5%)	3 (30%)
Hematology	18 (12%)	2 (20%)
Medicine	87 (59%)	5 (50%)
Surgery	23 (15,5%)	0 (0%)
Comorbidities (n, %)		
Chronic heart disease/HTA	77 (52%)	3 (30%)
Chronic kidney disease	31 (21%)	3 (30%)
Chronic lung disease	13 (9%)	2 (20%)
Chronic liver disease	9 (6%)	2 (20%)
Solid organ/Bone marrow transplant	8 (5%)	3 (30%)
Immunosuppression (n, %)	31 (21%)	6 (60%)
Source of BSI (n, %)		
Genitourinary	40 (27%)	1 (10%)
Central venous catheter	24 (16%)	3 (30%)
Intra-abdominal	27 (18%)	2 (20%)
Respiratory	5 (3%)	
Surgical site infection	9 (6%)	
SSTI	3 (2%)	
BJI	4 (3%)	
Endocarditis	4 (3%)	
Others	3 (2%)	
Unknown	12 (8%)	4 (40%)
Contaminant	17 (11%)	
CRP (mean +/- SD)	134 +/- 94	91 +/- 81
Creatinine (mean +/- SD)	138 +/- 149	108 +/- 80
WBC count (mean +/- SD)	16 +/- 22	28 +/- 45
delay (h) for BC positivity (median +/- SD)	12 +/- 27	36 +/- 11
30d mortality (n, %)	15 (10%)	4 (40%)

**Table 6:** Results of retrospective analysis of potential clinical impact of ePlex BCID assay, stratified by type of medical unit.

Potential therapeutic modification, No. (%)		None	Stop	De-escalation	Optimization	Escalation
**Based only on identification result of bacteria or yeast:**	**Total**	**99 (63%)**	**2 (1%)**	**22 (14%)**	**28 (18%)**	**7 (4%)**
	ICU	15 (65%)		2 (9%)	6 (26%)	
	Hematology	12 (60%)		3 (15%)	4 (20%)	1 (5%)
	Medicine	60 (65%)	2 (2%)	12 (13%)	13 (14%)	5 (5%)
	Surgery	12 (52%)		5 (22%)	5 (22%)	1 (4%)
**Based on identification of bacteria or yeast AND resistance results:**	**Total**	**87 (55%)**	**2 (1%)**	**27 (17%)**	**25 (16%)**	**17 (11%)**
	ICU	14 (61%)		3 (13%)	6 (26%)	
	Hematology	11 (55%)		4 (20%)	4 (20%)	1 (5%)
	Medicine	53 (58%)		14 (15%)	11 (12%)	14 (15%)
	Surgery	11 (48%)		6 (26%)	4 (17%)	2 (9%)

To measure the added value of early detection of antimicrobial resistance genes by ePlex BCID Panels compared to other rapid assays that only provide rapid identification results, we distinguished potential therapeutic modifications that would have been done for the 158 BSI, based only on identification result of bacteria or yeast or based on identification of bacteria or yeast and resistance results.

We also explored the added value of detecting antimicrobial resistance genes more rapidly (**Table 4** and **S2**). The presence of resistance genes would have led to modifications of treatment in 28/158 (18%) of the patients. In particular, escalation rate would have increased from 7/158 (4%) to 17/158 (11%) patients and the de-escalation rate from 22/158 (14%) to 27/158 (17%) patients.

Overall, PCR results would have led to an erroneous decision in 6/158 (4%) patients while absence of an ePlex result was considered a loss or a probable loss of opportunity in 28% of the patients. Moreover, additional measures would have been taken earlier in 18 patients due to the molecular result: infection control measures in 11 patients (9 ESBL-producing *Enterobacterales*, 1 MRSA, 17nbsp;*S. pyogenes* strain), 3 transesophageal echocardiography, 1 readmission, 2 catheter removals, and 1 reoperation (**Table S3**).

## Discussion

### High Performance for the Identification of Bacteria and Fungus Involved in BSI

As only a limited number of genera or species represent over 80% of BSI, molecular methods have been developed and implemented in several laboratories. Large syndromic multiplex PCR panels such as the ePlex BCID Panels are now able to provide identification and detection of clinically relevant pathogens and antimicrobial resistance genes in one to two hours (Altun et al., 2013; Banerjee et al., 2016; Fiori et al., 2016; Kim et al., 2016; Timbrook et al., 2017; Beckman et al., 2019; Huang et al., 2019; Oberhettinger et al., 2020). Gram stain is required prior to performing the test to choose the appropriate ePlex BCID Panel(s) and prevents the user from running the test on false-positive blood cultures. The list of targets present on the panels seems well designed, as only 9% of GP bacteria, 5% of GN bacteria, and 13% of fungi were not included on the panels in our study, while previous reports found between 2.2% (for the BCID-GP Panel only) and 6% of pathogens untargeted by the panels (Maubon et al., 2018; Huang et al., 2019; Carroll et al., 2020; Oberhettinger et al., 2020). Identification to the species level was obtained for 61% of GP bacteria, 90% of GN bacteria, and 87.5% of fungi. Identification to the genus level increased detection rate of GN pathogens to 93%, while 87 and 88% had been previously detected in other studies analyzing 125 and 33 positive blood cultures respectively (Huang et al., 2019; Oberhettinger et al., 2020). Identification to the genus level increased identification rate to 91% for the GP strains, mainly because putative contaminants such as Coagulase-negative *Staphylococcus* species, *Corynebacterium* and *Micrococcus* strains are only identified to the genus level, with the exception of *S. epidermidis* and *S. lugdunensis* species identified by the BCID-GP Panel. Three recent evaluations of 98, 94, and 1,297 positive blood cultures with GP bacteria, respectively, also accurately identified 91% of the strains to the species or genus level (Huang et al., 2019; Carroll et al., 2020; Oberhettinger et al., 2020). Importantly, the ePlex BCID-GP Panel permitted the distinction between *E. faecalis* and *E. faecium* species for 14 strains, allowing an earlier switch toward beta-lactam antibiotics if *E. faecalis* was identified (*i.e.* for 3/4 patients infected with *E. faecalis* strains in our study). The ePlex BCID-GP Panel also identifies GP rods belonging to the genera *Corynebacterium*, *Lactobacillus*, *Bacillus* (*B. subtilis* group and *B. cereus* group) and the anaerobic species *Cutibacterium acnes*, authorizing faster de-escalation of antibiotic therapy if a contaminant is identified. Recent comparison of the FilmArray^®^ and ePlex BCID Panel performances on 98 GP bacterial samples respectively showed 29 versus 68% identification of GP bacteria to the species level and 88 versus 91% identification to the genus level (Oberhettinger et al., 2020).

The eSensor^®^ technology employed by the ePlex is very sensitive as only 2/193 (1%) microorganisms (2 *P. aeruginosa* strains, Se = 75%) provided false negative results with the RUO cartridges. The Pan Gram-Positive target on the BCID-GN Panel is designed to detect bacteria belonging to the genera *Staphylococcus*, *Streptococcus*, *Enterococcus*, or to the *Bacillus cereus* group and *Bacillus subtilis* group. According to this definition, the Pan Gram-Positive target was not designed to detect the *C. aerofaciens*, *D. pneumosintes*, and *C. acnes* isolates seen in our study, but it should have detected the strain belonging to *Streptococcus anginosus* group and the *E. faecium* strain. Microscopic examination in these instances helped to triage the laboratory to test the BCID-GP Panel in parallel in all samples. A Pan *Candida* target is also present on the BCID-GN Panel, allowing the detection of main *Candida* species (*C. albicans*, *Candida glabrata*, *Candida parapsilosis*, and *Candida krusei*). The BCID-GP Panel includes the same Pan *Candida* target and a Pan Gram-Negative target detecting ~95% of GN bacterial genera (including but not limited to *Enterobacteriacea*e, *Acinetobacter* sp., *Bacteroides* sp., *Neisseria* sp., *Stenotrophomonas* sp., and *Pseudomonas* sp.). Specificity of almost all targets present on the BCID-GP Panel were excellent as no false positive targets were detected for the genus or species specific targets (Sp = 100%) and only two false-positive Pan Gram-Negative targets and two Pan *Candida* targets (Sp = 97%) each. Among the four false positive results that were obtained with the BCID-GN Panel, Gram-stain results could have helped to eliminate false-identification of *N. meningitidis* but not the other GN rods. However, after resolution of discrepancies and re-testing on FP and FN results on CE-IVD cartridges, we obtained a 100% sensitivity and specificity for targets of all panels. Importantly, the BCID-GN Panel also helped to identify 2 bacterial isolates that were missed in unsuspected polymicrobial samples as previously experienced (Huang et al., 2019). One *B. fragilis* strain was missed by standard culture. Gram stain had shown only a GPCP and anaerobic Columbia blood agar media was overwhelmed with *S. anginosus* group in culture. However, culture of the frozen aliquot on anaerobic selective media did allow recovery of *B. fragilis*. Likewise, one *S. maltophilia* strain was also missed in a *P. aeruginosa*-positive culture but recovered from frozen samples. Moreover, identification of a *C. glabrata* strain by BCID-FP panel in an aerobic bottle was confirmed only 24 h later by isolation of the strain in the anaerobic bottle.

We experienced 9% of polymicrobial samples for which performance of the ePlex BCID Panels could be of particular help. Complete agreement was observed for 88% of the 17 samples with positive detection of up to 7 targets in one sample, showing excellent performance of this multiplex technology. However, careful attention has to be paid when interpreting the results as the presence of one bacterium from a given species may mask another bacterium from the same genus. For example, despite concordant PCR results, one *Streptococcus* strain belonging to *mitis/oralis* group was not suspected because of the combined presence of a *Streptococcus* belonging to *anginosus* group.

### Rapid and Accurate Detection of Resistance Genes in BSI

Detection of antimicrobial resistance genes yielded no discrepant results in our study while other studies reported very few instances, for *mecA* and *vanA* genes only (Huang et al., 2019; Carroll et al., 2020; Oberhettinger et al., 2020). Data on antibiotic resistance genes would have led to modifications of antibiotic therapy in 18% of the patients either due to detection of a resistance gene (9.5%) or the absence of a resistance gene (8.2%). Rapid detection of resistance genes is of great importance to guide early adaptation of treatment (Banerjee et al., 2015; MacVane and Nolte, 2016; Angelis et al., 2020). Importantly, the BCID-GN Panel contains the *bla_CTX-M_* target, which allowed for the detection of 17 ESBL-positive *Enterobacterales* that would have led to treatment escalation in 9/14 (65%) patients infected with ESBL-carrying strains. Four out of the five patients already treated with effective antibiotics were in ICU wards. For GP pathogens, added value of antimicrobial resistance genes would have resulted in escalation or to de-escalation of treatment more often than with identification alone and reduced the use of expensive antibiotics such as daptomycin. Moreover, while resistance genes are not present on the BCID-FP Panel, identification of *C. krusei* or *C. glabrata* is important as both species have low susceptibility to fluconazole while identification of other *Candida* species could allow an early switch from caspofungin to fluconazole.

### Easy Workflow Is a Great Laboratory Benefit in the 24/7 Management of BSI

Finally, one of the main advantages of these panels is the ease of use of the cartridges and the platform allowing the panels to be tested on a 24/7 basis. It could be helpful to maintain equal performance and with a uniform time to result for BSI during the day and night shifts or during the weekends, as lab technicians trained in microbiology may not be available around the clock. Training of the personnel able to test samples on the ePlex requires only 1 h, with no pre-requisition of skilled microbiology, except for the reading of the Gram stain. Interpretation of the PCR results should be cautious and transmitted to an antibiotic stewardship team to allow adaptation of empiric antibiotic therapy more rapidly. After insertion of the blood culture bottles inside the Bactec™ incubators, with the assumption of a 2 h delay to run the test, mean time to results would have been 21 h in our study. Retrospective analysis for potential clinical impact highlighted that 45% of the patients could have benefited from rapid molecular results if transmitted to an antibiotic stewardship team and that absence of an ePlex BCID Panel result was a loss of opportunity in 28% of patients, mainly because adaptation of empiric treatment was delayed for at least one day or because toxic antibiotics were pursued for at least one additional day.

### Limitations of the Study

The main limitations of our study were that only a retrospective analysis of clinical impact and a small sample size prevented us from performing statistical analyses on stratified clinical results. Nevertheless, our data are in accordance with other clinical studies that have already proved rapid molecular panels help to decrease time to identification, time to effective therapy and time to escalation or de-escalation by 5 to 40 h (Banerjee et al., 2015; MacVane and Nolte, 2016; Timbrook et al., 2017). Panel-based rapid molecular testing is also important for good clinical practice as they allow a reduction in the use of broad-spectrum antibiotics and the treatment of contaminants, while showing variable impact among studies with regard to mortality, length of stay or costs (Banerjee et al., 2015; MacVane and Nolte, 2016; Pardo et al., 2016; Nasef et al., 2020). These panels with enlarged numbers of species and antimicrobial resistance genes detected should further increase the impact of rapid molecular diagnosis for patients with BSI. Moreover, the number of positive fungal blood cultures tested was too low to draw strong conclusions on the performance of the ePlex BCID-FP Panel regarding fungal detection and the impact on patient management in case of fungemia. This will require more broadscale studies including a larger number of fungemia episodes. The BCID-FP panel has shown over 96% sensitivity and 99.8% specificity for *Candida* targets, *Cryptococcus neoformans*, *C. gattii*, *Fusarium* spp., and *Rhodotorula* spp targets in a multicentric study of 141 clinical samples and 725 contrived samples (Zhang et al., 2020).

## Conclusion

In conclusion, the ePlex BCID Panels provide sensitive and reliable results with a hands on time reduced to 2 min and a high potential impact of the results on clinical decisions. Further studies with real time transmission of the results will be necessary to measure real impact on mortality or length of stay and to obtain medico-economic data about these panels.

## Data Availability Statement

The raw data supporting the conclusions of this article will be made available by the authors, without undue reservation.

## Ethics Statement

Ethical review and approval was not required for the study on human participants in accordance with the local legislation and institutional requirements. Written informed consent for participation was not required for this study in accordance with the national legislation and the institutional requirements.

## Author contributions

Conceptualization: PP, MM, and YC. Methodology: PP, MM, and YC. Investigation and formal analysis: SB, IA, IP, JB, ST, DM, MC, CR, PP, and YC. Data curation: SB, DM, ST, PP, and YC. Writing—original draft preparation: SB and YC. Writing—review and editing: all authors. Supervision: PP and YC. Funding acquisition: MM and YC. All authors contributed to the article and approved the submitted version.

## Funding

The study was funded by an internal innovation grant from Grenoble Alpes University Hospital and by internal funding of the laboratory. Part of the BCID kits were kindly provided by GenMark Diagnostics. The funder was not involved in the study design, collection, analysis, interpretation of data, the writing of this article or the decision to submit it for publication.

## Conflict of Interest

The authors declare that the research was conducted in the absence of any commercial or financial relationships that could be construed as a potential conflict of interest.
