# Supplementary material for: Evaluation of Microbiological Performance and the Potential Clinical Impact of the ePlex® Blood Culture Identification Panels for the Rapid Diagnosis of Bacteremia and Fungemia
Source: Front Cell Infect Microbiol. 2020 Nov 26;10:594951. doi: 10.3389/fcimb.2020.594951 (PMC7726344; doi:10.3389/fcimb.2020.594951)
Supplement: Supplementary file 1 [file Table_1.docx]

Supplementary Material

# Supplementary Tables

Table S1 : List of targets present in ePlex BCID-GP, BCID-GN and BCID-FP panels

| **BCID-GP** | **BCID-GN** | **BCID-FP** |
| --- | --- | --- |
| *Bacillus cereus*group | *Acinetobacter baumannii* | *Candida albicans* |
| *Bacillus subtilis*group | *Bacteroides fragilis* | *Candida auris* |
| *Corynebacterium* | *Citrobacter* | *Candida dubliniensis* |
| *Cutibacterium acnes* | *Cronobacter sakazakii* | *Candida famata* |
| *Enterococcus* | *Enterobacter*(non-*cloacae* complex) | *Candida glabrata* |
| *Enterococcus faecalis* | *Enterobacter cloacae*complex | *Candida guilliermondii* |
| *Enterococcus faecium* | *Escherichia coli* | *Candida kefyr* |
| *Lactobacillus* | *Fusobacterium*nucleatum | *Candida krusei* |
| *Listeria* | *Fusobacterium necrophorum* | *Candida lusitaniae* |
| *Listeria monocytogenes* | *Haemophilus influenzae* | *Candida parapsilosis* |
| *Micrococcus* | *Klebsiella oxytoca* | *Candida tropicalis* |
| *Staphylococcus* | *Klebsiella pneumoniae* | *Cryptococcus gattii* |
| *Staphylococcus aureus* | *Morganella morganii* | *Cryptococcus neoformans* |
| *Staphylococcus epidermidis* | *Neisseria meningitidis* | *Fusarium* |
| *Staphylococcus lugdunensis* | *Proteus* | *Rhodotorula* |
| *Streptococcus* | *Proteus mirabilis* |  |
| *Streptococcus agalactiae*(GBS) | *Pseudomonas aeruginosa* |  |
| *Streptococcus anginosus*group | *Salmonella* |  |
| *Streptococcus pneumoniae* | *Serratia* |  |
| *Streptococcus pyogenes*(GAS) | *Serratia marcescens* |  |
| **Resistance Genes** | *Stenotrophomonas maltophilia* |  |
| *mecA* | **Resistance Genes** |  |
| *mecC* | CTX-M |  |
| *vanA* | KPC |  |
| *vanB* | NDM |  |
| **Pan Targets** | VIM |  |
| Pan Gram-Negative | IMP |  |
| Pan *Candida* | OXA |  |
|  | **Pan Targets** |  |
|  | Pan Gram-Positive |  |
|  | Pan *Candida* |  |

Table S2: Focus on ePlex results of polymicrobial samples compared to SOC testing

| **Gram stain** | **Panel tested and Positive ePlex targets** | **SOC results** | **Concordance/Point of attention** |
| --- | --- | --- | --- |
| GPCP | BCID-GP: *Enterococcus, E. faecalis, Staphylococcus, S. aureus* | *E. faecalis, S. aureus* | Concordant |
| EB-GNR | BCID-GN: *E.coli, Proteus, P. mirabilis* | *E. coli, P. mirabilis* | Concordant |
| EB-GNR and GPCP | BCID-GP: *Enterococcus, E. faecium, Staphylococcus*, pan-GN BCID-GN: *P. aeruginosa*, panGP | *P. aeruginosa, E. faecium*, MR *S. haemolyticus* | Concordant |
| GPCP | BCID-GP: *Enterococcus, E. faecium, Streptococcus*, *vanA* | *E. faecium vanA*-positive, *S. mitis/oralis* group | Concordant |
| EB-GNR | BCID-GN: *E. coli, K. pneumoniae* (2 episodes of BSI) | *E. coli, K. pneumoniae* | Concordant |
| GPR | BCID-GP: No target detected | *A. neuii, V. parvula* | Pan GP target not detected (species not covered by pan GP target) |
| GNR and GPCP | BCID-GP: *Enterococcus, E. faecalis,* pan-GN BCID-GN: *Proteus, P. mirabilis*, pan-GP | *E. faecalis, P. mirabilis* | Concordant |
| EB-GNR and other GNR | BCID-GN: *K. oxytoca, Proteus, P. mirabilis, M. morganii* | *K. oxytoca, P. mirabilis, M. morganii* | Concordant |
| GNR and GPCP | BCID-GP: no GP target, pan GN BCID-GN: *B. fragilis, P. aeruginosa* | *B. fragilis, P. aeruginosa, C. aerofaciens, D. pneumosintes* | *P. aeruginosa* not detected in aerobic bottle with RUO cartridge, detected with CE-IVD cartridge. True negative of Pan GP target detected (species not covered) |
| EB-GNR and GPCP | BCID-GP: *Streptococcus, S. anginosus group*, pan-GN BCID-GN: *Proteus, P. mirabilis* | *P. mirabilis, S. anginosus group* | Pan-GP target not detected with RUO cartridge, detected with CE-IVD cartridge |
| GPR and GNR | BCID-GP: *C. acnes*, pan-GN BCID-GN: *P. aeruginosa* | *C. acnes, P. aeruginosa* | Pan-GP target not detected (species not covered by panGP target) |
| GPCP | BCID-GP: *Streptococcus, S. anginosus* group | *S. anginosus group, S. mitis/oralis* group | *S. mitis/oralis* not suspected with molecular results |
| EB-GNR and GPCP | BCID-GP: *Enterococcus, E. faecium*, pan-GN BCID-GN: *E. coli, bla_CTX-M_* | *E. faecium, ESBL-producing E. coli* | Pan-GP target not detected with RUO cartridge, detected with CE-IVD cartridge |
| GPCP | BCID-GP: *Streptococcus, S. anginosus* group, pan-GN BCID-GN : *B. fragilis*, pan-GP | *S. anginosus* group*, B. fragilis* (only after secondary culture of the frozen aliquot) | True positive |
| GNR | BCID-GN: *P. aeruginosa, S. maltophilia* | *P. aeruginosa, S. maltophilia* (only after secondary culture of the frozen aliquot) | True positive |
| Yeast | BCID-FP: *C. krusei, C. glabrata* | *C. krusei, C. glabrata* (*C. glabrata* not detected by culture in aerobic bottle) | True positive |

*GNR : Gram-negative rods ; EB : Enterobacteriaceae ; GPCC : Gram-positive cocci in cluster ; GPCP : Gram-positive cocci in pairs or chains*

Table S3: Results of retrospective analysis of potential clinical impact of ePlex BCID assay, stratified by Gram-stain

| **Potential therapeutic modification, No.(%) following…** | **None** | **Stop** | **De-escalation** | **Optimization** | **Escalation** | **Loss of chance** | | | | **Erroneous decision based on PCR result** | | | **Other impact** |
| --- | --- | --- | --- | --- | --- | --- | --- | --- | --- | --- | --- | --- | --- |
|  |  |  |  |  |  | **Yes** | **P** | **PN** | **No** | **Yes** | **P** | **No** |  |
|  |  |  |  |  |  |  |  |  |  |  |  |  |  |
| **… identification results only : Total** | **99 (63%)** | **2 (1%)** | **22 (14%)** | **28 (18%)** | **7 (4%)** |  |  |  |  |  |  |  |  |
| GP | 28 (48%) | 2 (3%) | 11 (19%) | 15 (26%) | 2 (3%) |  |  |  |  |  |  |  |  |
| GN | 58 (74%) |  | 8 (10%) | 9 (12%) | 3 (4%) |  |  |  |  |  |  |  |  |
| PM (GN+GP) | 8 (62%) |  | 1 (8%) | 2 (15%) | 2 (15%) |  |  |  |  |  |  |  |  |
| FP | 5 (56%) |  | 2 (22%) | 2 (22%) |  |  |  |  |  |  |  |  |  |
| **… identification and resistance results: Total** | **87 (55%)** | **2 (1%)** | **27 (17%)** | **25 (16%)** | **17 (11%)** |  |  |  |  |  |  |  |  |
| GP | 26 (45%) | 2 (3%) | 14 (24%) | 13 (22%) | 3 (5%) | 17 | 5 | 1 | 35 | 1 | 1 | 56 | 2 TEE, 2 infection control measures |
| GN | 48 (62%) |  | 10 (13%) | 8 (10%) | 12 (15%) | 12 | 5 |  | 62 | 2 |  | 76 | 9 infection control measures ; 1 readmission ; 1 TEE ; 1 catheter removal ; 1 reoperation |
| PM (GN+GP) | 8 (62%) |  | 1 (8%) | 2 (15%) | 2 (15%) | 2 | 1 |  | 10 | 2 |  | 12 | none |
| FP | 5 (56%) |  | 2 (22%) | 2 (22%) |  | 2 |  |  | 7 |  |  | 9 | 1 TEE, 1 catheter removal |

TEE : transesophageal echocardiography ; P : Probably ; PN : Probably not
